# c-Jun N-Terminal Kinase Signaling Inhibitors Under Development

**DOI:** 10.5487/TR.2008.24.2.093

**Published:** 2008-06-01

**Authors:** Sun-Young Han

**Affiliations:** Drug Discovery Division, Korea Research Institute of Chemical Technology, 19, Sinseongno, Yuseong-gu, Daejeon, 305-343 Korea

**Keywords:** JNK, Inhibitors, SP-600125, CC-401, AS-602801, CEP-1347, D-JNKI-1

## Abstract

Targeting protein kinases has been active area in drug discovery. The c-Jun N-terminal kinases (JNKs) have also been target for development of novel therapy in various diseases, since the roles of JNK signaling in pathological conditions were revealed in studies using *jnk*-deficient mice. Small molecule inhibitors and peptide inhibitors are identified for therapeutic intervention of JNK signaling pathway. SP-600125, an anthrapyrazole small molecule inhibitor for JNK with high potency and selectivity has been widely used for dissecting JNK signaling pathway. CC-401 is the first JNK inhibitor that went into clinical trial for inflammation and leukemia. Inhibitor for mixed lineage kinase (MLK), CEP-1347 also negatively regulates JNK signaling, and tried for potential use in Parkinson’s disease. Cell-permeable peptide inhibitor D-JNKI-1 is being developed for the treatment of hearing loss. The current status of these JNK inhibitors and safety issue is discussed in the minireview.

## INTRODUCTION

One of the most active areas in drug development is targeting protein kinases. Out of 518 protein kinases identified from human genome project (Manning *et al.*, 2002), quite a few kinases are known to be involved in signal transduction pathway. And dysregulation of the signaling pathway due to the overexpression, mutation, or deletion of protein kinases are related to pathophysiological process. In practical point of view for drug development, kinases are easy to inhibit using small molecules. The ATP binding pocket shared by all protein kinases is binding site for small molecules and serves as a pharmacophore. As results of efforts for drug development targeting kinases, several drugs are on the market. Since the first kinase inhibitor on the market imatinib (Gleevec) for treatment of leukemia, several kinase inhibitors are launched recently. These include drugs targeting epidermal growth factor receptor (EGFR), vascular endothelial cell growth factor receptor (VEGFR), and second generation *bcr-abl* inhibitors. Also small molecules targeting other kinases are under active development by pharmaceutical companies. JNK signaling pathway is one of the molecular targets for therapeutic purpose.

JNK signaling pathway has been a focus of attention for therapeutic target for its diverse roles in cellular processes (for review, refer to Davis, 2000; Weston and Davis, 2002). JNK was initially purified as a protein activated by a variety of stress signals (Kyriakis and Avruch, 1990). Other group independently identified JNK for its affinity to c-Jun N-terminal domain (Hibi *et al.*, 1993). Later this kinase was molecularly cloned and found to be a member of the mitogen-activated protein kinase (MAPK) group. Like other MAPK subfamily, JNK signaling is transmitted by way of three-kinase signaling module comprised of MAPK, MAP2K, and MAP3K (Fig. 1). Members of the JNK family are encoded by three genes (*jnk1*, *jnk2*, and *jnk3*), which are alternatively spliced to yield ten isoforms (Barr and Bogoyevitch, 2001; Bennett *et al.*, 2001).

Also the role of JNK pathway in pathological condi-

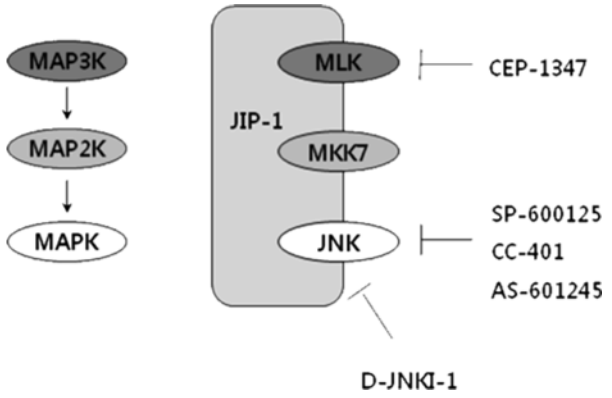

**Fig. 1.** JNK signaling cascade and inhibitors. JNK signaling pathway is activated by three-kinase signaling module composed of MAPK, MAP2K, and MAP3K, like other MAPK family. For JNK family, the kinase module is composed of JNK1, JNK2, and JNK3 for MAPK, MKK4 and MKK7 for MAP2K, and diverse MAP3Ks including MLKs. The components of the signaling modules are organized by binding to scaffold protein. JIP-1 is a scaffold protein organizing MLK, MKK7, and JNK. JNK signaling inhibitors are indicated for their site of action. CEP-1347 is an ATP-competitive inhibitor for MLK activity. SP-600125, CC-401, and AS-601245 are small molecule inhibitors for JNK activity. D-JNKI-1 is a cell-permeable peptide inhibitor containing the 20 peptide sequence of JIP-1. Figure is adapted from Borsello and Forloni (2007).

tion has been demonstrated in animal models including *jnk*-deficient mice. Studies of JNK signaling pathway suggested JNK can be a therapeutic target for diseases including inflammation, neurodegenerative diseases, cancer, obesity, and diabetes. Manning and Davis wrote an excellent review for this topic (Manning and Davis, 2003). Here in this review, current development status of JNK signaling inhibitors will be described along with safety issues of drugs targeting JNK.

## INHIBITORS OF JNK SIGNALING CASCADE

JNK signaling pathway is known to be very important in various pathological conditions. However, there is very short list for small molecule inhibitors under active development targeting JNK protein directly compared to other kinases (Table 1). And most of the JNK inhibitors reported in patent literatures are byproducts from search of p38 MAPK inhibitors (Harper and LoGrasso, 2001; Manning and Davis, 2003). These may reflect the difficulty of finding selective inhibitors for JNK. Here I will describe the first JNK selective inhibitor SP-600125 and other direct JNK inhibitors under active development. Additionally, MLK inhibitor CEP-1347 and peptide inhibitor D-JNKI-1 will be discussed (Fig. 1). The JNK signaling inhibitors are listed in Table 1 together with development status and the name of the company developing the agents.

***SP-600125.*** SP-600125 is a selective JNK inhibitor with chemical structure of anthra[1,9-cd]pyrazol-6-(2H)-one (Fig. 2). This anthrapyrozole compound was identified using high-throughput biochemical screen by Celgene corporation (previously known as Signal Pharmaceuticals) (Bennett *et al.*, 2001). SP-600125 is reversible ATP-competitive inhibitors, with $IC_{50}$ of 40 nM for JNK1, 40 nM for JNK2, and 90 nM for JNK3. SP-600125 shows selectivity more than 20-fold over 17 other kinases tested. SP-600125 has 300-fold selectivity for JNK compared with extracellular signal-regulated kinases (ERKs) and p38 MAPKs in the MAPK family. However, SP-600125 inhibits the upstream of JNK and p38 MAPK signaling pathway, which are MKK4 (MAPK kinase 4), MKK7, MKK3, and MKK6, with $IC_{50}$ values of between 0.4 and 5 $\mu$M. Thus, the interpretation of the experimental results with high concentration of SP-600125 should be done cautiously. Furthermore, report by Bain *et al.* (2003) indicated that SP-600125 may not be a selective inhibitor for JNK protein, contradicting previous reports. $IC_{50}$ values for JNK1$\alpha$1 and JNK2$\alpha$2 were weak at 0.1 mM ATP (5.8 $\mu$M and 6.1 $\mu$M, respectively). And some other kinases were inhibited to a greater extent than JNK.

When SP-600125 was first reported in 2001 as a JNK inhibitor (Bennett *et al.*, 2001), this compound became instantly popular in studies dissecting the roles of JNK signaling pathway. Unlike ERK or p38 MAPK

**Table 1.** JNK signaling inhibitors

| Agent | Company | Target | Status | Therapeutic group |
|---|---|---|---|---|
| SP-600125 | Celgene | JNK | | |
| CC-401/JNK-401 | Celgene | JNK | Phase II | Myeloid Leukemia, Inflammatory disease |
| AS-601245 | Merck Serono | JNK | Preclinical | Cystic fibrosis |
| CEP-1347/KT-7515 | Cephalon | MLK | | Parkinson's disease |
| D-JNKI-1/AM-111/XG-102 | Auris Medical | JNK | Phase I/II | Hearing loss<br>Myocardial infarction<br>Ischemic stroke |

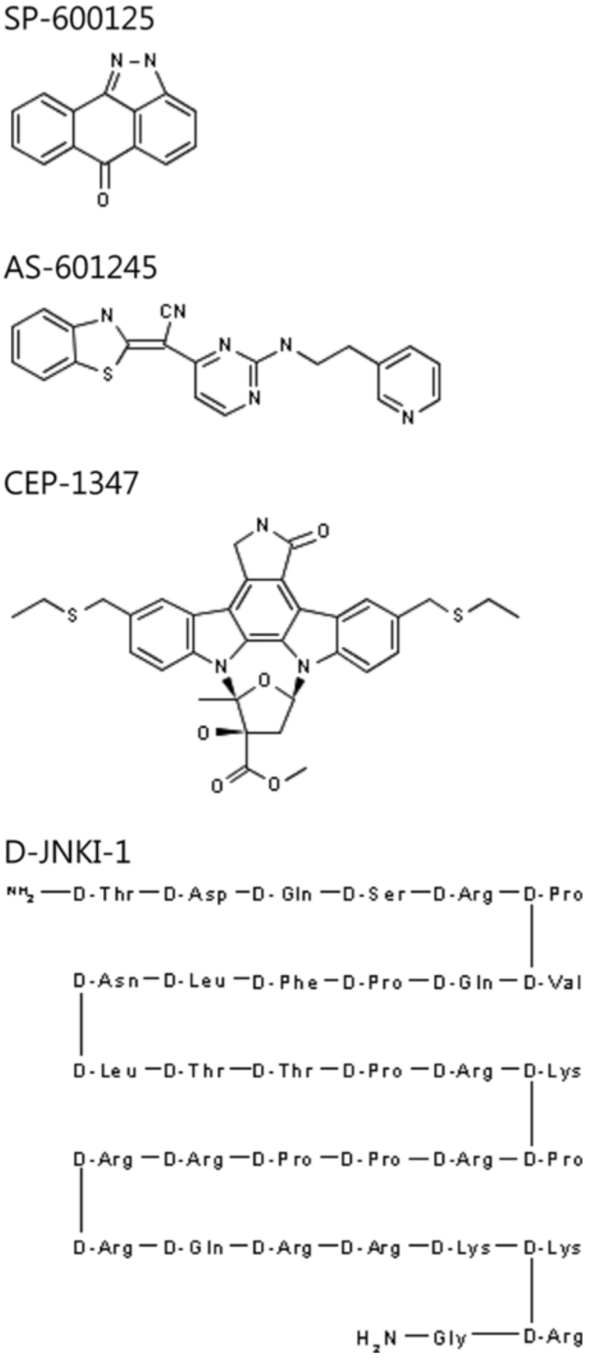

**Fig. 2.** Chemical structures of JNK pathway inhibitors.

pathway, there were no small molecule inhibitors for JNK pathway. To dissect ERK signaling pathway, MEK1/ MEK2 inhibitor PD98059 and U0126 had been used (Favata *et al.*, 1998). And SB202380 was applied for the study of p38 MAPK signaling pathway at that time (Lee *et al.*, 2000; Salituro *et al.*, 1999). To investigate the JNK pathway, no small molecules were available at that time. Only approach available was the use of dominant negative form of JNK, which required transfection process. With the identification of SP-600125 at 2001, this compound was used extensively in combination of dominant negative protein approach in studying cellular biology of JNK pathway.

The potential of SP-600125 in disease application was first shown by the study of Han *et al.* (2001). To induce arthritis in animal model, Lewis rats were immunized with adjuvant. After 8 days, SP-600125 (30 mg/ kg/day) was injected subcutaneously for 6 days. SP-600125 affected very modestly on the inflammation as shown by foot pad swelling. However, it provided marked protection from joint destruction as indicated by radiographs of rat joint bones. This study suggested that JNK-inhibitory compounds could be clinically effective in the treatment of rheumatoid arthritis. This study was followed by other reports of SP-600125 application in various animal disease models such as tumor xenograft (Ennis *et al.*, 2005), ischemia/reperfusion injury (Ishii *et al.*, 2004; Wang et al., 2007), bacterial otitis media in guinea pig (Furukawa *et al.*, 2007), and smoke inhalation injury in rats (Syrkina *et al.*, 2007).

***CC-401.*** CC-401, also known as JNK-401, is highly selective and ATP-competitive inhibitor of JNK. CC-401 is the first JNK inhibitor which entered into clinical trial by Celgene Corporation. This compound is in phase I trial for the treatment acute myeloid leukemia and inflammatory disease. However, details regarding clinical trials and chemical structure are not disclosed yet. CC-401 inhibits all three forms of JNK isoforms with 25 to 50 nM of Ki (Ma *et al.*, 2007) and has more than 40-fold selectivity for JNK over other related kinases (e.g., p38 MAPK, ERK, and IκB kinase 2).

Two studies were reported for the potential use of CC-401 during transplantation procedures. During the organ transplantation, the organs get damaged from cold ischemic storage followed by warm reperfusion. Rapid JNK activation is observed after ischemia/reperfusion procedures. To prevent injury of the organ during transplantation and to address the role of JNK pathway after transplantation, selective JNK inhibitor CC-401 was used. Pretreatment of CC-401 increased survival after transplantation in both orthotopic rat liver transplantation model (Uehara *et al.*, 2004) and rat hepatic ischemia/ reperfusion model (Uehara *et al.*, 2005). Recent study investigated the potential use of CC-401 in progressive kidney disease by blocking renal fibrosis and tubular apoptosis (Ma *et al.*, 2007). Gavage administration of CC-401 blocked JNK activation and suppressed renal fibrosis and tubular epithelial cell apoptosis in rat unilateral ureteral obstruction model. Furthermore, Ma *et al.*

(2007) showed that nonredundant role of JNK1 in tubular cell apoptosis. Only mice with JNK1 deletion not JNK2 deletion showed significant reduction in tubular apoptosis. CC-401 was also reported to be effective in rat model of inflammatory disease called glomerulonephritis (Flanc *et al.*, 2007). CC-401 treatment suppressed glomerular and tubulointerstitial damage in immune-induced renal injury. Taken the studies together, treatment of CC-401 may be useful with therapeutic benefit, thus the safety data from ongoing phase I clinical trial will be expected.

***AS-601245.*** From the screening of compound library for JNK3 inhibitors, (benzothiazol-2-yl) acetonitrile derivatives were identified as a potent and selective JNK1, JNK2, and JNK3 inhibitors (Gaillard *et al.*, 2005). Further optimization of the compound led to the discovery of AS-601245 (Fig. 2). AS-600245 has $IC_{50}$ of 70 to 220 nM against JNK isoforms and it exhibited 10- to 20-fold selectivity over c-Src, c-Raf, and CDK2 (cyclin-dependent kinase 2). AS-601245 can be administered per oral route, and is under active development by Merck Serono.

*In vivo* effect of AS-601245 has been evaluated in mouse model of rheumatoid arthritis (Gaillard *et al.*, 2005), myocardial ischemia/reperfusion in rats (Ferrandi *et al.*, 2004), neuronal protection after cerebral ischemia in gerbils (Carboni *et al.*, 2004, 2007). Since *jnk3*-deficient mice exhibited dramatic protection from neuronal apoptosis induced by excitotoxicity (Yang *et al.*, 1997), it is considered good strategy to use JNK inhibitor for the purpose of neuroprotection. At a dose of 80 mg/kg, AS-601245 reduced neurites damage by 67% and decreased astrocytes activation by 84% (Carboni *et al.*, 2007). Also as neurobehavioral measure, AS-601245 improved long-term memory.

***CEP-1347.*** CEP-1347, also known as KT-7515 (for structure, see Fig. 2) is a semisynthetic derivative of K-252a, a natural product indolocarbazole of the bacterium Nocardiosis species (Kaneko *et al.*, 1997). At first, CEP-1347 was characterized to have neurotrophic properties (Kaneko *et al.*, 1997). And then it was identified to have inhibitory activity against JNK signaling pathway, but JNK was not a direct target of CEP-1347 (Maroney *et al.*, 1998). Later, the same group identified MLK family as a target of CEP-1347 (Maroney *et al.*, 2001). MLK family belongs to MAP3K that regulates JNK activity, and is consisted of MLK1, MLK2, MLK3, dual leucine zipper kinase, and leucine zipper kinase. CEP-1347 is ATP-competitive inhibitor with $IC_{50}$ values ranging from 23 to 51 nM.

CEP-1347 was developed focusing its activity on blocking cell death of motorneuron, therefore potential use in Parkinson's disease. To mimic human Parkinson's disease in animal model, neurotoxin called MPTP (1-methyl-4-phenyl tetrahydropyridine) is widely used to destroy nigrostriatal dopaminergic neurons (Langston, 1996). Administration of MPTP activated JNK pathway and application of CEP-1347 blocked JNK signaling (Saporito *et al.*, 2000). Also in this MPTP-induced animal model, CEP-1347 protected dopaminergic neurons from apoptosis (Saporito *et al.*, 1999). Based on the encouraging results in the animal model, clinical trials of CEP-1347 to delay disability in early Parkinson's disease were conducted by pharmaceutical company called Cephalon. To everybody's disappointment, treatment of Parkinson's disease with CEP-1347 was not effective in terms of delaying disease progression (Parkinson Study Group, 2007). According to the Parkinson study group who conducted clinical trial and comments by Ahlskog (Ahlskog, 2007) and Waldmeier *et al.* (Waldmeier *et al.*, 2006), this negative study gave us a few lessons. First, we still don't know enough about cell death mechanism in neurodegenerative diseases. Therapeutic intervention of JNK pathway may be compensated by other mechanisms. This compensation may make CEP-1347 ineffective in Parkinson's disease. Second, current animal model (MPTP-induced neurotoxicity) may not reflect human Parkinson's disease properly. Although, MPTP model provided insight into symptomatic treatment of Parkinson's disease, this model may not be able to predict for delaying progression of Parkinson's disease. A small molecule called TCH346 developed for Parkinson's disease with different mechanism showed good activity in MPTP-induced animal model. However, TCH346 failed to show efficacy in human. This can be an another example for the lack of correlation between MPTP model and human Parkinson's disease (Waldmeier *et al.*, 2006).

***D-JNKI-1/AM-111/XG-102.*** There are efforts identifying JNK inhibitors other than small molecule approach. D-JNKI-1 is a peptide inhibitor of JNK activity under active development. D-JNKI-1 is based on the peptide sequence of its scaffold protein called JIP-1 (JNK interacting protein-1) protein (Bonny *et al.*, 2001). There is JNK-binding domain (JBD) in JIP-1 protein, and transfection of 20 amino acid sequences from JBD inhibited JNK activity, presumably by blocking access to many of its substrates by a competitive mechanism (Barr *et al.*, 2002; Bonny *et al.*, 2001). This 20 amino acid peptide was further modified to make it cell-permeable. Covalent linkage of 10-amino acid sequence of TAT protein from HIV (human immunodeficiency virus) with this peptide enabled intracellular delivery (Schwarze *et al.*, 1999). Furthermore, the peptide was synthesized with all-D retro-inverso form, which is composed of D-instead of L-amino acids and synthesized in reverse order (Fig. 2). Although this switch from L-enantiomer decreased the efficacy of JNK-inhibitory effect, half-life of the peptide was markedly increased due to the protection from proteolytic cleavage.

D-JNKI-1 has property of potent activity inside the cells compared with *in vitro* biochemical activity. $IC_{50}$ of D-JNKI-1 is 1 mM *in vitro* biochemical JNK activity assay, whereas 1 nM of D-JNKI-1 is sufficient for protection of pancreatic β-cell apoptosis induced by IL-1β by 50% (Bonny *et al.*, 2001). Another group also reported inhibitory effect of peptide sequence derived from JIP-1 protein (Barr *et al.*, 2002). Barr *et al.* showed that the shorter size (11-amino acid) of JBD domain retained the inhibitory activity. Approach of linking TAT sequence was also used successfully for this smaller size peptide by another group (Guan *et al.*, 2006).

D-JNKI-1 was evaluated for neuroprotective effects in ischemic stroke model in rodents (Borsello *et al.*, 2003; Hirt *et al.*, 2004). D-JNKI-1 peptide is expected to block neuronal apoptosis induced by excitotoxicity during cerebral ischemia. This cell-penetrating peptide showed powerful protection (> 90%), and remarkably D-JNKI-1 was administered 6~12 hour after the onset of ischemia. This study indicated that neuronal death can be blocked 6~12 hour after the onset of stroke, establishing proof of principle for JNK pathway as a target for therapeutic intervention. Additionally, there are reports for the application of D-JNKI-1 for myocardial ischemia-reperfusion (Milano *et al.*, 2007) and use of similar JNK inhibitory peptide to diabetes (Kaneto *et al.*, 2004).

For practical point of view, there are limitations of peptide inhibitor in human therapy: cost and toxicity (Borsello and Bonny, 2004). The cost of producing peptide molecule is very expensive compared with small molecule inhibitor and systemic administration of peptide may be toxic when used in high dosage. Borsello and Bonny (2004) suggested restricting the use of peptide inhibitors in small organs, including eyes and ears to circumvent these problems.

For this reason, D-JNKI-1 is being developed for the treatment of hearing loss. Hearing loss come from cell death of auditory hair cells and auditory neurons caused by noise, infection and ototoxic drugs. The strategy was to block hair cell death by D-JNKI-1-mediated JNK inhibition. Wang *et al.* (2003) demonstrated that application of D-JNKI-1 prevented nearly all hair cell death and permanent hearing loss in guinea pig ototoxicity model. Furthermore, D-JNKI-1 was effective when applied 12 hours after the sound trauma in animal model (Wang *et al.*, 2007). Based on the effect of D-JNKI-1 on hearing loss in animal model, this peptide is in clinical trial for hearing loss by Auris Medical (Suckfuell *et al.*, 2007) and designated as orphan drug. The granting of orphan drug status gives advantage in developing drugs for rare diseases by way of tax reductions and marketing exclusivity for an extended time.

## SAFETY OF JNK SIGNALING INHIBITORS

During the course of drug development, evaluation of toxicological safety is important part of preclinical development. Also in clinical development, assessing safety is the main purpose of Phase I and Phase II clinical trials. When it comes to signaling pathway inhibitors, the toxicity manifested can be two kinds. One is from the unique characteristic of the specific chemical. In this case, we just need to stop the development of the specific chemical and find other agents. The other kind is mechanism-based toxicity, i.e. the one coming from the inhibition of the signaling pathway. For JNK signaling inhibitor, the toxicity may be manifested by the inhibition of cellular process mediated by JNK signaling. If toxicity from JNK pathway inhibition is a major problem, we may need to reconsider JNK pathway as a therapeutic target or find a way to avoid toxicity like local administration. Until now, three agents CC-401, CEP-1347, and D-JNKI-1 went into clinical trials. The safety data from the studies will be discussed in this chapter.

Our knowledge regarding clinical studies of CC-401 is limited due to the lack of publications of the data. Just from the press release, it is known that double-blind, placebo-controlled phase I study of ascending single intravenous doses are completed. The results from MLK inhibitor CEP-1347 in clinical studies by Parkinson Study Group are found in a few publications (Parkinson Study Group, 2007; Parkinson Study Group, 2004). Oral administration of CEP-1347 was reported to be generally safe and well tolerated in both healthy volunteers and Parkinson's disease patients. Cell-permeable peptide D-JNKI-1 was evaluated for its safety in patients with acute acoustic trauma after exposure to firecrackers on New Year's Eve (Suckfuell *et al.*, 2007). Intratympanic administration of D-JNKI-1 was generally safe and well-tolerated. Although a total of 13 adverse events were reported, the majority involving tinnitus, it seemed to be unrelated to the medication.

It seems too early to tell general safety of JNK signaling inhibitors based on the current clinical data. Although safety of CEP-1347 is confirmed in several clinical studies, caution should be taken to extrapolate the results to other JNK inhibitor. Since CEP-1347 does not inhibit JNK directly, direct JNK inhibitor may show different symptoms in terms of adverse effects. In case of D-JNKI-1, it was administered locally within the ears, thus systemic effect was not assessed in the phase I/II study (Suckfuell *et al.*, 2007).

## CONCLUDING REMARKS

In this review, current status of drug development for JNK signaling inhibitors is discussed. Novel therapeutic strategy using neuroprotective effects of JNK signaling inhibitor was highly expected, since protection from neuronal apoptosis in *jnk3*-deficient mice were quite dramatic (Yang *et al.*, 1997). Therefore, failure of translating this research into the therapy of Parkinson's disease using CEP-1347 was quite a disappointment (Parkinson Study Group, 2007). However, lack of efficacy for Parkinson's disease using MLK inhibitor CEP-1347 does not necessarily mean that JNK inhibitors are not applicable to other disorders accompanying neuronal cell death. Efforts should be continued for development of novel therapies using JNK inhibitors in diseases involving neuronal apoptosis. The case of D-JNKI-1 is quite encouraging that investigators found a niche in hearing loss for disease involving neuronal apoptosis. The research progress of CC-401 and AS-601245 during the next few years will be very exciting.

## Abbreviations

JNK: c-Jun N-terminal kinase
ATP: adenosine 5′-triphosphate
MLK: mixed lineage kinase
EGFR: epidermal growth factor receptor
VEGFR: vascular endothelial cell growth factor receptor
MAPK: mitogen-activated protein kinase
MAP2K/MKK: MAPK kinase
MAP3K: MAPK kinase kinase
MEK: MAPK-ERK kinase
ERK: extracellular signal-regulated kinase
Ki: inhibitory constant
IKK: IκB kinase
IC_50_: concentration of compound to achieve 50% inhibition
CDK: cyclin-dependent kinase
MPTP: 1-methyl-4-phenyl tetrahydro-pyridine
JBD: JNK-binding domain
JIP: JNK-interacting protein
IL-1β: interleukin-1β
